# Identification of Sesame Genomic Variations from Genome Comparison of Landrace and Variety

**DOI:** 10.3389/fpls.2016.01169

**Published:** 2016-08-03

**Authors:** Xin Wei, Xiaodong Zhu, Jingyin Yu, Linhai Wang, Yanxin Zhang, Donghua Li, Rong Zhou, Xiurong Zhang

**Affiliations:** Key Laboratory of Biology and Genetic Improvement of Oil Crops of the Ministry of Agriculture, Oil Crops Research Institute of the Chinese Academy of Agricultural SciencesWuhan, China

**Keywords:** sesame, genome comparison, genomic variation, SNP, Indel, landrace

## Abstract

Sesame (*Sesamum indicum* L.) is one of the main oilseed crops, providing vegetable oil and protein to human. Landrace is the gene source of variety, carrying many desire alleles for genetic improvement. Despite the importance of sesame landrace, genome of sesame landrace remains unexplored and genomic variations between landrace and variety still is not clear. To identify the genomic variations between sesame landrace and variety, two representative sesame landrace accessions, “Baizhima” and “Mishuozhima,” were selected and re-sequenced. The genome sequencing and *de novo* assembling of the two sesame landraces resulted in draft genomes of 267 Mb and 254 Mb, respectively, with the contig N50 more than 47 kb. Totally, 1,332,025 SNPs and 506,245 InDels were identified from the genome of “Baizhima” and “Mishuozhima” by comparison of the genome of a variety “Zhongzhi13.” Among the genomic variations, 70,018 SNPs and 8311 InDels were located in the coding regions of genes. Genomic variations may contribute to variation of sesame agronomic traits such as flowering time, plant height, and oil content. The identified genomic variations were successfully used in the QTL mapping and the black pigment synthesis gene, *PPO*, was found to be the candidate gene of sesame seed coat color. The comprehensively compared genomes of sesame landrace and modern variety produced massive useful genomic information, constituting a powerful tool to support genetic research, and molecular breeding of sesame.

## Introduction

Sesame (*Sesamum indicum* L.) is one of the main oilseed crops, providing vegetable oil and protein to human (Weiss, 2000; Wei et al., 2013). It is also one of the most ancient oilseed crops, starting its domestication from the wild progenitor *S*. *malabaricum* in the Indian subcontinent about 5000 years ago (Fuller, 2003; Pathak et al., 2015). Its high oil quality and benefit to human health has earned it the poetic label “queen of oilseeds” (Johnson et al., 1979). Because of the popular of sesame among consumers, the market demand for sesame seeds is growing rapidly and the planting area of sesame has almost doubled in the last fifty years (Faostat, 2015).

A widely grown sesame variety in China, “Zhongzhi13,” has been *de novo* sequenced recently (Wang et al., 2014b), which had opened the door of genome research and genomic selection breeding of sesame. The genome sequencing revealed that sesame has a small diploid genome, which was estimated to be 350 Mb. In total, 274 Mb draft genome of “Zhongzhi13” was successfully assembled and 27,148 protein-coding genes were predicted. Although the genome sequencing of “Zhongzhi13” had been completed, the lack of enough genomic information still limited the genetic improvement of sesame. Comprehensive and exhaustive identification of the genomic variations in sesame genome are urgent for this important oilseed crops.

Because of the bottleneck occurs during the artificial, landraces are considered to carry many agriculturally desirable alleles that are not contained in varieties (Doebley et al., 2006; Cavanagh et al., 2013). Landraces could provide useful genes for genetic improvement of crops. The agriculturally valuable alleles identified in landraces genome can be easily transferred to commercial varieties by introgression breeding (Zeven, 1998; Zamir, 2001). A lot of superior alleles from landrace have been discovered and used in the crop breeding. For example, the superior allele of the rice “green revolution” gene *sd1* was identified from a rice landrace “Deo-geo-woo-gen” (Spielmeyer et al., 2002). One allele of *GS2* in the rice landrace “Baodali” could increase 40% thousand seed weight and 14% yield of plant (Hu et al., 2015). And an allele of *qHSR1* in the maize landrace “Ji1037” showed high resistance of the systemic disease heat smut (Zuo et al., 2015).

As a worldwide grown crop, the landraces of sesame have diverged to various genotypes to adapt different environment and climate. Previous studies revealed that sesame landraces had a large scale of diversity in both phenotype and genotype (Zhang et al., 2012; Yue et al., 2013; Wang et al., 2014a; Wei et al., 2015). And multiple loci that related to several agronomic traits had been identified in sesame landraces, including oil and protein content (Li et al., 2014), plant height (Ding et al., 2013), disease resistance (Zhang et al., 2012), drought tolerance (Li et al., 2013), waterlogging tolerance (Zhang et al., 2014), seed coat color (Zhang et al., 2013). These research implied that large number of desirable alleles were carried in sesame landraces and largely needed to be discovered. However, genome of sesame landraces still have not been explored and genomic variation have not been detected.

To investigate the genome of sesame landraces, two landraces (“Baizhima” and “Mishuozhima”) from South China and East China, respectively, were selected and *de novo* sequenced in the present research. Previous genetic research showed these two landraces had far relationship to “Zhongzhi 13” (Wang et al., 2014a; Wei et al., 2014). Each landrace was sequenced approximately 70-fold coverage of the predicted sesame genome. We compared the genomes of landraces and variety and discovered a large number of SNPs and InDels in the genomes. Large-effect SNPs and InDels were detected in some genes related to important agronomic traits of sesame. Identification of genomic variations in sesame landraces will provide massive valuable information for genetic research and molecular breeding of sesame in future.

## Materials and methods

### Sampling and genome sequencing

**Figure 1:**
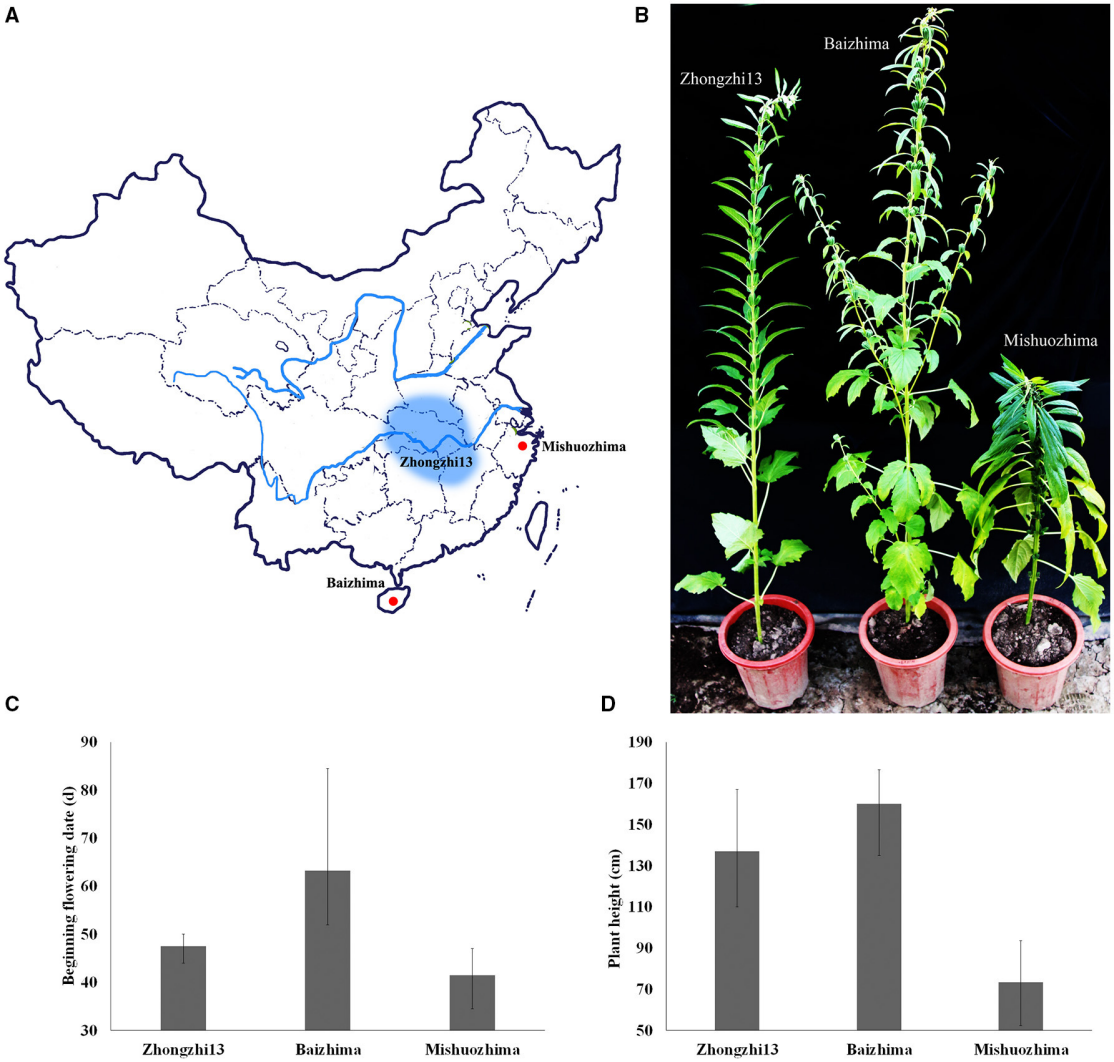
**Geographic origin and phenotypes of “Baizhima,” “Mishuozhima,” and “Zhongzhi13”**. **(A)** Geographic origin; **(B)** Individuals in the end of flowering stage; **(C)** Beginning flowering date; **(D)** Plant height.

Two representative sesame landraces (Table 1 and Figure 1), “Baizhima” and “Mishuozhima,” preserved at the China National Gene Bank, were selected and self-pollinated for six generation. Thirty-two SSR markers (two primers in each linkage group of sesame genome) were selected to detect the heterozygosity of the two accessions (Table S1). The samples were planted in Wuhan in the summer of 2014 and fresh leaves were collected from 4 weeks seedlings. Total genomic DNA was extracted by using the DNeasy Plant Mini Kit (Qiagen, USA). Library construction was done as described (Huang et al., 2009). A total of 49 Gb high-quality reads were generated using Illumina Hiseq2500 platform, representing a raw coverage of approximately 70-fold coverage of the genome for each landrace, using an estimated genome size of 350 Mb for sesame genome (Wang et al., 2014b). The DNA sequencing data were deposited in the European Nucleotide Archive (http://www.ebi.ac.uk/ena/data/view/PRJEB8078).

**Table 1:** **List of the landraces sequenced in this study**.

**Accession name**	**Group**	**Original producing area in China**	**Latitude**	**Longitude**	**Seed color**	**Thousand seed weight (g)^a^**	**Capsule number per axil**	**Ridge number of capsule**	**Branchiness**
Baizhima	Landrace	Qiongzhong, Hainan	109.50E	19.05N	White	1.52 ± 0.07	1	8	Multi-branched
Mishuozhima	Landrace	Dongyang, Zhejiang	120.23E	29.26N	Black	2.74 ± 0.10	3	4	Unbranched
Zhongzhi13	Variety	Yangtze river basin	NA	NA	White	3.15 ± 0.08	3	4	Unbranched

a *Investigated from 2011 to 2013 in Wuhan of China*.

### Sequence assembling and comparison

The genomes of two landrace were assembled by the SOAPdenovo2 package (version 2.04) (Luo et al., 2012). The reads were split into *k*-mers (k was 61 and 55 for “Baizhima” and “Mishuozhima,” respectively) from the short-insert size libraries. The gaps in the contigs were excluded by Gapcloser (version 1.12). The N50 length of the final assembly was calculated without all small contigs of < 200 bp. Contig numbers of different length were counted by in house Perl script. MUMmer was used in the anchoring of the assemblies to the sesame reference genome (Kurtz et al., 2004), and the diffseq program in the EMBOSS package was used in the calling of sequence variants (Rice et al., 2000). The reference genome “Zhongzhi13,” mRNA, protein sequences, repeat sequences, transposons types, and GFF file were downloaded from the Sinbase database (Wang et al., 2014b). Transposons in “Baizhima” and “Mishuozhima” were identified by the software RepeatMasker (Version 3.3.0, http://repeatmasker.org) with the default parameters. The repeat sequence library used in the transposons identification was downloaded from http://www.girinst.org. Homologous sequence used in the transposons alignment including LG1 (11920217 to 12241908) in “Zhongzhi13,” contig tarseq_824_000001_1 of “Baizhima” genome and contig tarseq_2333_000001, tarseq_2333_000002, tarseq_6067_000001, tarseq_749_000001, tarseq_749_000002 in “Mishuozhima” genome. Transposons types in “Baizhima” and “Mishuozhima” genome were annotated by aligning with the reference genome. Circos (Krzywinski et al., 2009) was used to construct the diagram of genome variations of all 16 linage groups in sesame. Phylogenetic tree of the three sesame accessions was constructed by the software PHYLIP (Felsenstein, 1989). Genome of *Utricularia gibba* (Ibarra-Laclette et al., 2013) was used as out-group.

Gene structure of the two genomes was predicted by the software FGeneSH (Salamov and Solovyev, 2000). *Arabidopsis* genome and gene annotation were download from the TAIR database (www.arabidopsis.org). All genes in “Zhongzhi13” genomes were aligned with *Arabidopsis* genes by BLAST (Altschul et al., 1997) and gene function was annotated by the *Arabidopsis* genes (*e* < 10^−5^ and protein sequence identity rate more than 70%). Then the best matched *Arabidopsis* genes were used for the annotation of sesame genes. Genes in “Baizhima” and “Mishuozhima” were aligned with that from “Zhongzhi13” genome and annotated by *Arabidopsis* genes. SNPs and InDels in coding regions were identified by self-customized Perl script from the genome comparison files generated by the diffseq program. Synonymous and non-synonymous SNPs were also districted by self-customized Perl script. And dN/dS rate of each gene was calculated from the number of synonymous and non-synonymous SNPs. KEGG annotation of the genes with dN/dS ratio > 1 was performed with the annotation of the reference genome. The KEGG pathway of the genes were clustered on the KEGG website (http://www.kegg.jp/). The potential effects of the SNPs and InDels were predicted based on the annotation of “Zhongzhi13” genome with the GFF files. The *de novo* assemblies and genome-wide analysis of all the coding variants are available at the Sesame Haplotype Map Project database (http://www.ncgr.ac.cn/SesameHapMap).

### QTL mapping

Recombinant inbred lines (RILs) were developed from a cross between “Zhongzhi13” and “Mishuozhima” followed by self-fertilization to F6, containing 550 individuals. The population was developed in the experimental field at Oil Crops Research Institute in Wuhan, Hubei Province. For genotyping, total genomic DNA was isolated from leaf tissues using the DNeasy Plant Mini Kit (Qiagen, USA). About four thousand SSR markers were designed from the sesame genome (Wei et al., 2014). All of them were used in the screening of the parent DNA and 400 polymorphism SSR primers were selected. Then the polymorphism SSRs were used in the QTL mapping for all F6 RILs. Six QTLs of sesame coat color were identified. To fine mapping the major QTL, 80 SSRs in the QTL region were developed and screened the RILs (Table S2). Seed coat color of the mature seeds was recorded for RILs. The color was recognized by CR-400 Chroma Meter (Konica Minolta, Japan) and recorded as standard color evaluation formula (L*a*b*).

### qRT-PCR

Developing seeds of “Zhongzhi13” and “Mishuozhima” were collected from the capsules at 5, 8, 11, 14, 17, and 20 days after flowering. All seeds were carefully collected on the ice and put into liquid nitrogen immediately. Total RNAs of the seeds were extracted by the EASYspin Plus kit (Aidlab, China) following the manufacturer's instructions. Any remaining DNA in the RNA was treated with DNaseI. The RNA was reverse transcribed by HiScript II 1st Strand cDNA Synthesis kit (Vazyme, China) with oligo (dT23) primer. Gene-specific primers and probes were designed for all candidate genes. The primers and probes used in the gene amplification were listed in Table S3. The qRT-PCR experiments were performed with Premix Ex Taq (Takara, Japan) on the CFX384 Real-Time System (Bio-Rad, USA). The actin7 gene (SIN_1006268) was used as an internal control.

## Results

### *De novo* assembly of sesame landrace genomes

Two landraces (Table 1), “Baizhima” and “Mishuozhima,” which from South China and East China were selected and *de novo* sequenced. These two landraces were quite different from the variety “Zhongzhi 13” in several important agronomic traits, such as plant height, branchiness, seed size, seed number per capsule, disease resistance, and flowering date (Figure 1). “Zhongzhi13” is a high yielding variety, the major characters including unbranched, three capsules per axil, high plant height, and white seed. “Mishuozhima” is a typical semi-dwarf germplasm with black seed. “Baizhima” is a branched and late flowering germplasm with small white seed. Both SSR and SNP analysis showed significant genetic differentiation of the three sesame accessions (Wang et al., 2014a; Wei et al., 2014). Although sesame is mainly self-pollinated, out-crossing rate in natural environment was between 4 and 23% (Pathirana, 1994; Sun et al., 2015). These two landraces were strictly maintained by self-pollination for six generation to obtain highly homologous genomes. Heterozygosity of the samples were detected by 32 SSR markers (Table S1). No heterozygous locus was identified in both two landraces, indicating highly homozygous of the samples.

Genomic DNA was extracted from seeding of 4 weeks for each landrace. The DNA libraries were constructed with an insert size of 300 bp and sequenced by the Illumina Hiseq2500 platform. Approximately 2.5 Gb data were generated for each sample, covering 70-fold genome size of sesame. SOAPdenovo was used to assemble the genomes (Luo et al., 2012), resulting in a draft genome of 267 and 254 Mb for “Baizhima” and “Mishuozhima,” respectively. The draft genome size was similar to that of “Zhongzhi13” (274 Mb; Wang et al., 2014b). In total, 73,395 and 49,167 contigs were assembled for “Baizhima” and “Mishuozhima” (Table S4 and Supplementary Figure S1), with the contig N50 size of 47.2 and 47.8 kb, respectively. These contigs were aligned with the reference genome “Zhongzhi13” using MUMmer software (Kurtz et al., 2004). Most sequence of “Baizhima” and “Mishuozhima” genome (82 and 86%, respectively) could be identified in the reference genome except the repetitive sequences.

Gene structure in “Baizhima” and “Mishuozhima” was predicted and the genes were compared with that from the reference genome (Supplementary Figure S2). In total, 77% of the coding genes (19,263 and 19,221 of “Baizhima” and “Mishuozhima,” respectively) were annotated for each genome of “Baizhima” and “Mishuozhima” (Table S5). Among them, 17,029 genes were *Arabidopsis*-homologous genes, which might be conservative genes.

Phylogenetic tree of the three genomes was constructed by PHYLIP (Felsenstein, 1989) and *Utricularia gibba* genome was used as out-group (Supplementary Figure S3). *U. gibba*, the tiny genome size plant, was reported to be the closest species of sesame in all sequenced species (Ibarra-Laclette et al., 2013). The phylogenetic analysis clearly showed that “Baizhima” and “Mishuozhima” has a closer relationship than “Zhongzhi 13,” indicating that plenty of variations might be discovered from genome comparison of the variety and landraces.

### Landscape of genome variations between sesame landrace and variety

**Figure 2:**
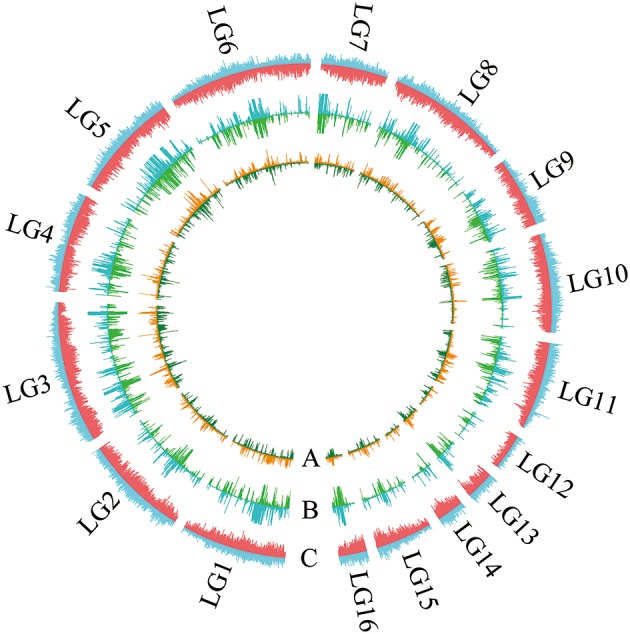
**Landscape of the genome variation in “Baizhima” and “Mishuozhima**. ” Distribution of **(A)** SNPs, **(B)** Indels, and **(C)** Transposons in the genomes. The upside and downside of each circle show genome variations of “Baizhima” and “Mishuozhima,” respectively. The linkage groups of sesame genome are indicated.

**Figure 3:**
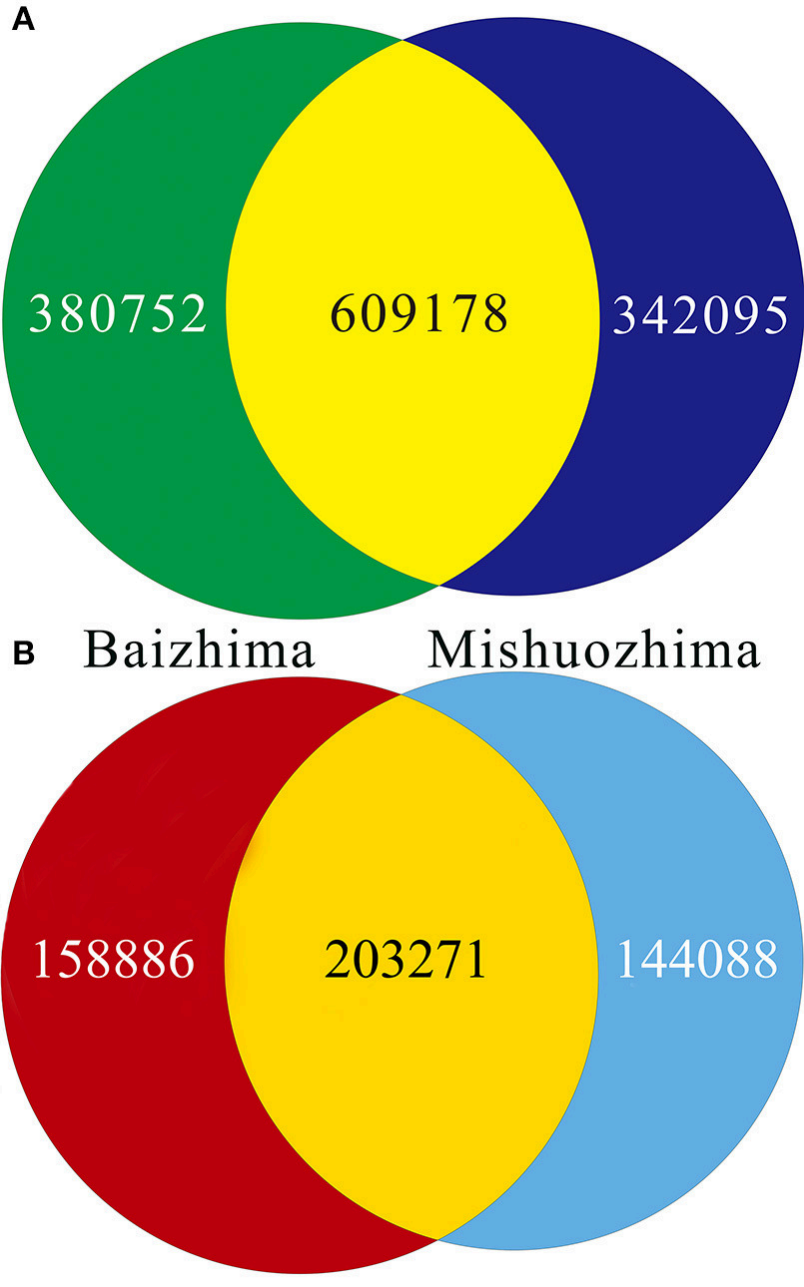
**Venn diagrams of SNPs and InDels in genomes of “Baizhima” and “Mishuozhima.” (A)** SNPs; **(B)** Indel.

All contig of “Baizhima” and “Mishuozhima” genomes were aligned against the reference genome, “Zhongzhi13.” A large number of genome variations were identified, mainly including SNPs, InDels and transposons (Figure 2). Totally, we identified 1,332,025 SNPs, varying from 1.15 SNPs to 8.65 SNPs in 1 kb among the 16 linkage groups of the two landrace genome (Table 2). In average, it is about 4.9 SNPs in 1 kb of the two genomes. Approximately 46% (609,178) of the SNPs were shared by the two landrace genomes (Figure 3A). The 12 nucleotide substitution types ranged from 34,549 to 162,568 (Table 3 and Supplementary Figure S4A). Among the them, G:A, A:G, C:T, and T:C substitutions were the most common SNPs, in line with the result in the previous research of sesame (Wang et al., 2014a).

**Table 2:** **Summary of SNPs and InDels rate in LGs of sesame genome**.

**LGs**	**LG length (bp)**	**SNPs**				**InDels**			
		**Number in Baizhima**	**Rate in Baizhima (SNPs/kb)**	**Number in Mishuozhima**	**Rate in Mishuozhima (SNPs/kb)**	**Number in Baizhima**	**Rate in Baizhima (InDels/kb)**	**Number in Mishuozhima**	**Rate in Mishuozhima (InDels/kb)**
1	18577331	81285	4.38	65543	3.53	26939	1.45	23969	1.29
2	18500646	49964	2.70	51768	2.80	18627	1.01	18429	1.00
3	24928530	159130	6.38	156471	6.28	52441	2.10	51437	2.06
4	17356267	83379	4.80	82983	4.78	29392	1.69	29372	1.69
5	18898134	163548	8.65	145640	7.71	60563	3.20	54592	2.89
6	25289714	91586	3.62	100394	3.97	38274	1.51	39506	1.56
7	11725536	51181	4.36	53081	4.53	17002	1.45	17849	1.52
8	21523998	69132	3.21	59884	2.78	24673	1.15	22233	1.03
9	12411895	47237	3.81	39152	3.15	18759	1.51	16006	1.29
10	17245970	57009	3.31	56988	3.30	23251	1.35	22572	1.31
11	15446199	57290	3.71	53268	3.45	21503	1.39	19525	1.26
12	6373461	7353	1.15	9978	1.57	3182	0.50	3861	0.61
13	5050363	16447	3.26	21632	4.28	6100	1.21	7347	1.45
14	4882680	10683	2.19	6763	1.39	4330	0.89	3044	0.62
15	10047770	21933	2.18	21060	2.10	8212	0.82	8018	0.80
16	4963887	22773	4.59	26632	5.37	8909	1.79	9599	1.93
Average	14576399	61870.63	3.89	59452.31	3.81	22634.81	1.44	21709.94	1.40

**Table 3:** **Number of all SNPs and InDels types in “Baizhima” and “Mishuozhima” genomes**.

**SNP type**	**Number in Baizhima**	**Number in Mishuozhima**
G->C	35865	34549
C->G	36187	34810
T->G	42004	40487
A->C	42001	40580
C->A	42432	40911
G->T	42753	41194
A->T	53026	51056
T->A	53303	51326
T->C	158750	151978
A->G	159231	153484
G->A	161805	155200
C->T	162568	155655
**InDel type (bp)^a^**	**Number in Baizhima**	**Number in Mishuozhima**
1	43771	45022
2	23096	23654
3	11852	12105
4	13026	13017
5	11501	12024
6	8311	8365
7	7612	7795
8	8531	8663
9	8625	8851
10	7666	7698

a *Only 1 to 10 bp InDels are listed*.

In total, 506,245 InDels (including structural variations) were detected in the two genomes, containing 203,271 shared InDels (Figure 3B). Distribution of InDels was also not parallel in the LGs, the highest InDel rate (LG5, 3.20 InDels in 1 kb) was six times of the lowest one (LG12, 0.50 InDels in 1 kb) (Table 2). As expect, small InDels were dominant of all InDels and single-base and two-base InDels were the most common InDels (Table 3 and Supplementary Figure S4B).

Among all SNPs and InDels, more than 90% were found to locate in intergenic and intronic regions of sesame genome (Table 4). Large percent of genome variations in intergenic and intronic regions of genome was also reported in other species, such as rice, maize, soybean, grape, and tomato (Chia et al., 2012; Huang et al., 2012; Di Genova et al., 2014; Lin et al., 2014; Zhou et al., 2015). Since variations of coding regions usually generate functional change of proteins and might affect plant growth and development.

**Table 4:** **Summary of genomic location of SNPs and InDels in “Baizhima” and “Mishuozhima” genome**.

**Location**	**SNPs**		**InDels**	
	**Baizhima**	**Mishuozhima**	**Baizhima**	**Mishuozhima**
5′ UTR	8976	9064	3110	3124
CDS	51326	51550	5594	5897
3′ UTR	8677	9245	2564	2724
Intron	92710	94795	27022	27547
mRNA	161689	164654	38290	39292
Intergenic	828241	786583	323867	308067
Total	989930	951237	362157	347359

We noticed that almost all kind of SNPs and InDels in “Baizhima” genome were more than that in “Mishuozhima” genome (Supplementary Figure S4), implying that “Mishuozhima” might have a closer relationship of “Zhongzhi13.” This result was in accordance with the geographic origin and phenotypes of the three accessions. “Mishuozhima” and “Zhongzhi13” are cultivated in the regions of similar latitude in China. Both “Mishuozhima” and “Zhongzhi13” are unbranched, early flowering and have three capsules per axil, while “Baizhima” is much branched, late flowering and have only one capsule per axil.

Transposable elements are one of the most common genomic variations in plants (Lisch, 2013). To compare transposable elements in the genomes of landrace and variety, the longest contig (tarseq_824_000001) in LG1 of “Baizhima” genome was selected and transposons were analyzed in the homologs region of the three genomes (Supplementary Figure S5). In this region, we found that long terminal repeats, including Gypsy-like and Copia retrotransposon elements were the most common transposons (Table S6).

### Alteration of coding DNA sequence in sesame landrace genomes

Genes were announced in the two genomes and compared with the reference genome. 70,018 SNPs (51,326 in “Baizhima” and 51,550 in “Mishuozhima”) and 8311 InDels (5594 in “Baizhima” and 5897 in “Mishuozhima”) were identified in coding regions of all genes (Supplementary Figure S6). Effects of the SNPs in coding regions were investigated and 33,385 synonymous SNPs and 36,633 non-synonymous SNPs in 9263 genes (34% of all sesame genes) were identified. Non-synonymous SNPs existed in 7404 genes (5577 in “Baizhima” and 5553 in “Mishuozhima”), indicating potential functional divergence of these genes.

We calculated the non-synonymous-to-synonymous ratio of the SNP data set. Compared with the ratio for total sesame genes (1.14 in “Baizhima” and 1.12 in “Mishuozhima”), the non-synonymous-to-synonymous ratio (dN/dS) for the *Arabidopsis*-homologous genes (0.90 in “Baizhima” and 0.91 in “Mishuozhima”) was significantly lower, indicating strong functional conservation of the gene set in evolution. Among them, 106 well-characterized genes related to flowering and 67 genes involved in lipid metabolism pathway were focused on specially (Table S7), which showed lower non-synonymous-to-synonymous ratio (0.57 and 0.39, respectively). The result suggested that lipid metabolism genes were more conservative than flowering genes in sesame.

Based on dN/dS > 1, we identified 2274 genes might be positive selected genes (Table S8). Among them, 886 genes had more than 10 SNPs (Table S9), indicating rapid divergence in protein coding regions. These genes included plenty of lipid metabolism genes, disease resistant genes, and flowering related genes. Among them, disease resistance genes were over-represented, suggesting the genes were undergoing strong natural and artificial selections. Similar phenomenon was also observed in the genome comparison of varieties or landraces in rice, maize, and grape (Springer et al., 2009; Li S. C. et al., 2012; Di Genova et al., 2014). KEGG pathway enrichment analysis of these genes were performed and these genes were founded to be involved in most KEGG pathways (Supplementary Figure S7), indicating that most pathways in sesame might have been selected during the domestication and artificial selection. The result showed that these genes were enriched in the carbohydrate metabolism and signal transduction pathways. Since both carbohydrate metabolism and signal transduction pathways are associated with plant abiotic stress such as salt, drought, and cold (Zhu, 2002; Baena-Gonzalez et al., 2007), the enrichment of the genes in these pathways might reveal the positive selection of abiotic stress tolerance for sesame.

Large-effect genomic variations which leading the loss function of genes and might significantly affect the agronomic traits were detected in “Baizhima” and “Mishuozhima” genomes. The large-effect SNPs causing premature to stop codon, start codon and stop codon changes were detected in 1276 genes in the genome of landraces (Table S10). While the large-effect InDels including frameshift InDels and InDels in start codon and stop codon were identified in 1932 genes in “Baizhima” and “Mishuozhima” genomes (Table S11). In total, 2627 large-effect SNPs were found in “Baizhima” and “Mishuozhima” genomes. Large-effect SNPs detected in “Mishuozhima” genome (1652) was much more than that in “Baizhima” genome (975). And the type of large-effect SNPs were also quite different between “Baizhima” and “Mishuozhima” genomes (Supplementary Figure S8). SNPs causing premature sites to be stop codons were dominant in “Baizhima” genome while SNPs which leaded start codon to be non-start codon was the most common ones in “Mishuozhima” genome. Large-effect genomic variations were found in the functional genes that related to disease resistant, flowering, plant development and lipid metabolism (Table S12). The identified large-effect SNP and InDels in crops can facilitate discovery and cloning of candidate genes related to the domestication and improvement (Yu, 2013).

### Genomic variations of the gene related to agronomic traits

**Figure 4:**
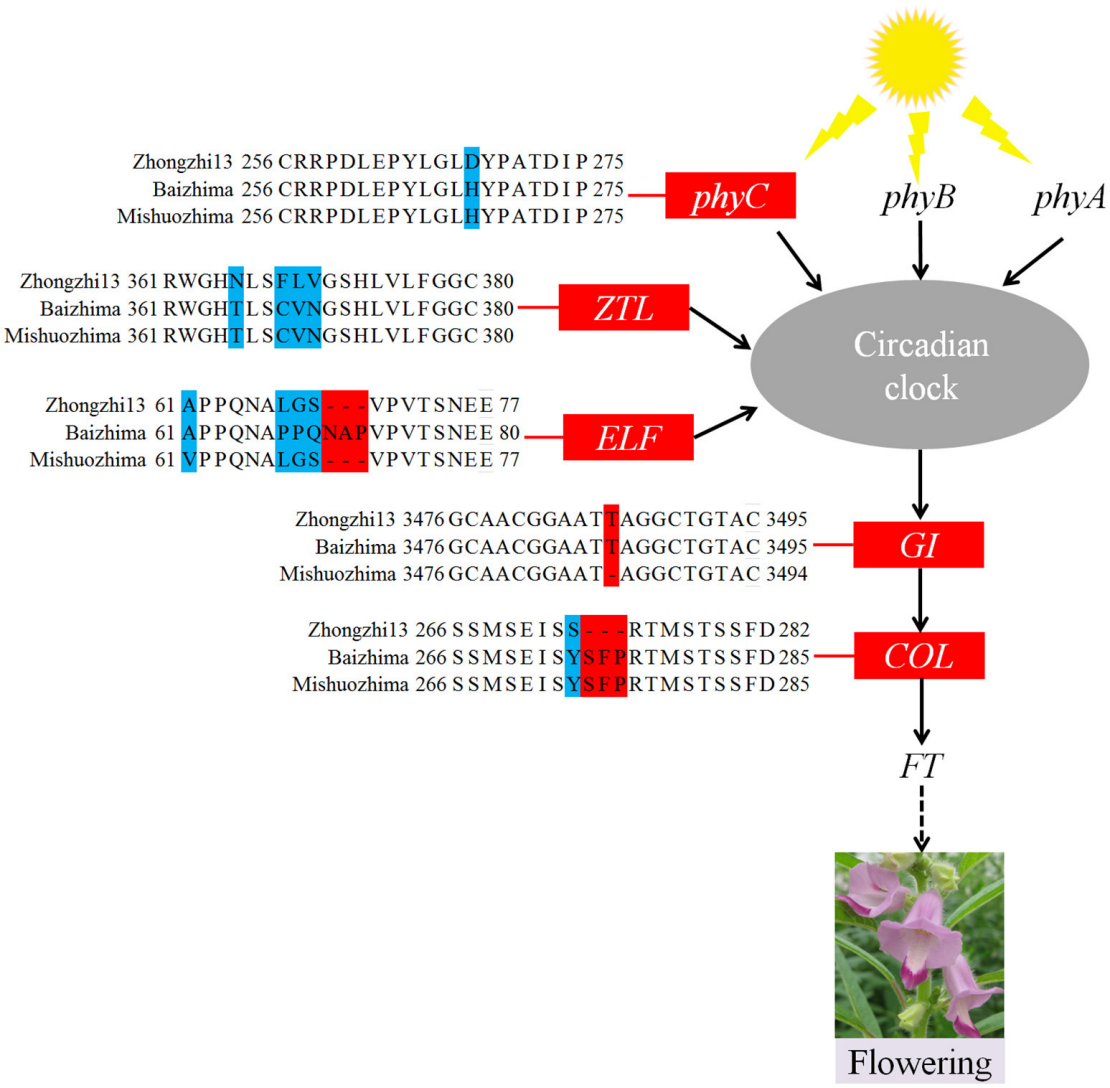
**Variations in photoperiod genes of sesame**. In *phyC, ZTL, ELF*, and *COL*, amino acid changes resulting from SNPs and InDels are shown. In the *GI* gene, a 1 bp insertion was found in “Zhongzhi13” and “Mishuozhima.”

As a short day plant, sesame usually flowers early in short day-light condition. However, sesame has been brought worldwide and flowers in extremely different day-light conditions. The flowering mechanism of the photoperiod way of sesame is largely remaining explored. Flowering date of “Zhongzhi13,” “Baizhima,” and “Mishuozhima” showed greatly diverse (about 45, 60, and 38 days in the summer of Wuhan in China). Here, we showed comparison of several photoperiod genes in the three sesame cultivars (Figure 4). It is clearly revealed that multiple polymorphisms existed in the photoperiod genes, including *phyC, ZTL, FLF, GI*, and *COL* (Putterill et al., 1995; Fowler et al., 1999; Somers et al., 2000; Wang and Deng, 2004; Song et al., 2009). *GI* is the key gene which receives signals from the circadian clock and transfers the information to *COL* (Putterill et al., 1995). One loss-of-function insertion in *GI* was found in “Zhongzhi13” and “Mishuozhima,” resulting unfunctional *GI* in the two accessions. The loss-of-function protein caused invalid of circadian clock system in sesame and flowering promoting of “Zhongzhi13” and “Mishuozhima” in long-day condition. The highly diversity of the photoperiod genes in sesame provides convincing explanation to the flowering date divergence of sesame. And the natural and artificial selection of the photoperiod genes spurred sesame expanding globally after its domestication in India (Bedigian, 2003, 2010).

Plant height decides the plant architecture and contributes to the sesame yield (Wang et al., 2016). Because high-stem varieties tends to lodging at the ripening stage, decreasing plant height of modern varieties is one of the major targets in sesame breeding. “Mishuozhima” is a typical dwarf landrace. Its plant height is about half of that of “Zhongzhi13” in different environment (Figure 1). Interestingly, a frameshift deletion was found in *GA20ox1* in “Mishuozhima” genome, but was not detected in “Baizhima” and “Zhongzhi13” genomes. *GA20ox1* is the famous “Green revolution” gene which leading the decrease of plant height and substantial increase of yield in rice (Spielmeyer et al., 2002). Mutations in *GA20ox1* also cause semi-dwarf phenotypes in *Arabidopsis* and barley (Jia et al., 2009; Barboza et al., 2013). This loss-of-function mutation in *GA20OX1* might be the key reason of the dwarf phenotype of “Mishuozhima.”

Oil content of sesame has been significantly selected such that varieties produce much more fatty acid than landraces. Oil content of “Zhongzhi13” (58%) is significantly higher than that in “Baizhima” (48%) and “Mishuozhima” (50%). Lipid metabolism genes in sesame were annotated based on homology analysis of the genes in acyl lipid pathways in *Arabidopsis*. Of these genes, large effect genes were found in *LTP, WRI1, ACNA*, and *LOX2*. Copy number of *LTP* and *LOX2* had been reported intensely affect oil content of sesame (Wang et al., 2014b). The genome-wide association study revealed that *ACNA* was associated with the content of stearic acid and peanut acid in sesame (Wei et al., 2015). And mutation of *WRI1* was found to cause an 80% reduction in seed oil content of *Arabidopsis* (Li X. et al., 2012). These genes might have been artificial selected in the sesame breeding and contributed to the improvement of seed oil concentration.

### Validation and utilization of the genomic variations

To validate if the genomic variation could be used in the genetic research, we used it in the QTL mapping of sesame seed coat color. Seed coat color is one of the most significant characters of sesame. The color of mature sesame seeds is diverse, varying from black, intermediate colors to white. Compared with white seeds, black sesame seeds usually have less oil but more protein and lignin (Zhang et al., 2013). In East Asia, black sesame seeds and its products are more popular among the consumers. Seed coat color was reported to be related to biochemical properties, antioxidant content, and disease resistance (Shahidi et al., 2006; El-Hamid Sayid El-Bramawy et al., 2008; Kanu, 2011). Previous studies indicated that sesame seed coat was controlled by one or two genes (Gutierrez et al., 1994; Zhang et al., 2013).

**Figure 5:**
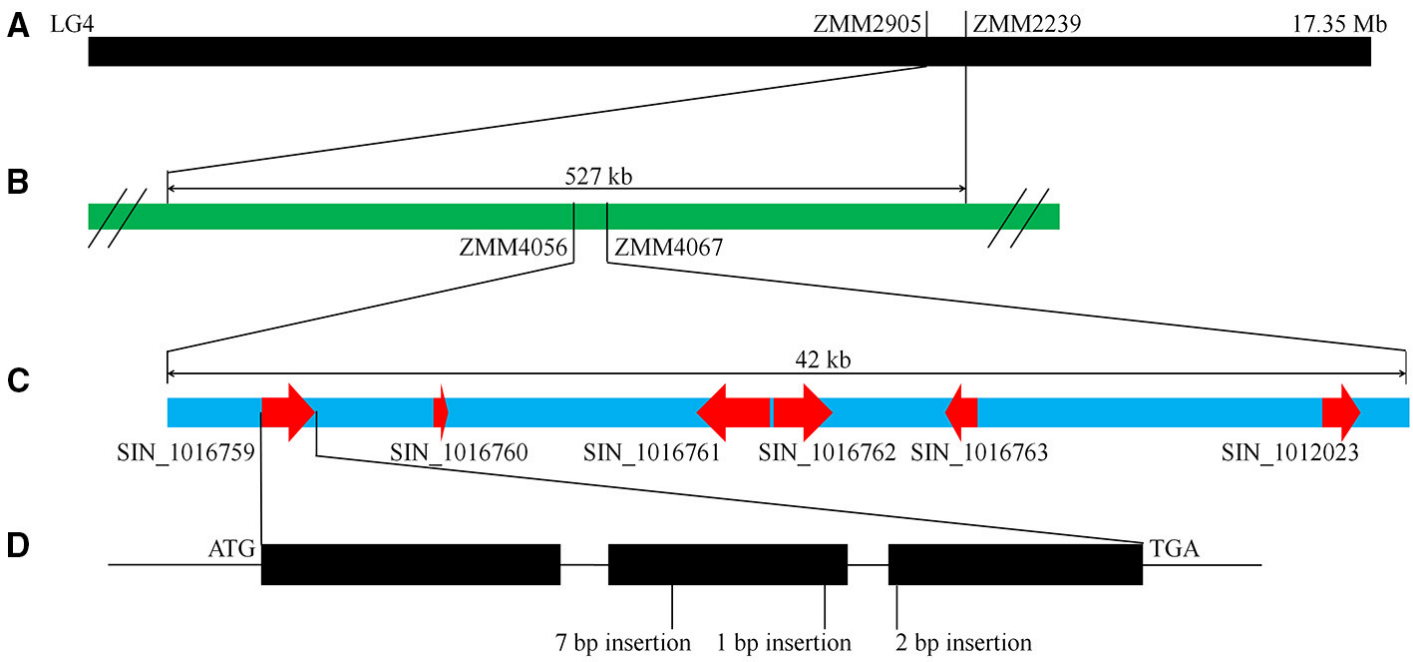
**QTL mapping and gene identification of seed coat color. (A)** A major QTL for seed coat color was identified in LG4. **(B)** Fine mapping of the QTL. **(C)** Predicated genes in the QTL region. **(D)** Frameshife InDels in SIN_1016759.

As mentioned above, “Zhongzhi13” is a white seed variety which “Mishuozhima” is a black seed landrace. To identify the major QTLs that control this trait, we previously constructed a F6 recombinant inbred lines (RILs) population derived from the cross between “Zhongzhi13” (white seed) as the recurrent parent and “Mishuozhima” (black seed) as the donor parent. One major QTL, which was accounted for 66.5% of the phenotypic variations, was detected between the markers ZMM2905 and ZMM2239 (527 kb) on LG4 (Figure 5). To fine mapping the locus and clone the gene for seed coat color, 80 SSR markers were designed based on the genome sequence of the two parents and were used to screen the population in the QTL region (Table S2). The candidate block of the QTL was sharped into 42 kb, between ZMM4056 to ZMM4067. Six candidate genes were identified in this region.

Sequence of candidate genes in “Zhongzhi13” and “Mishuozhima” was aligned. Frameshift InDels were found in one gene (SIN_1016759) of “Zhongzhi13,” suggesting SIN_1016759 might be the key gene related sesame seed coat color. Then qRT-PCR was performed to investigate the gene expression of the candidate genes. Gene specific primers and probes were designed and used in the qRT-PCR (Table S3). Developing seeds from 5 to 20 days of “Zhongzhi13” and “Mishuozhima” were collected and expression of the genes was detected. The qRT-PCR results showed that all candidate genes did not expressed in all seeds of “Zhongzhi13” and only SIN_1016759 expressed in the seeds from 11 to 20 days of “Mishuozhima.” Homology analysis revealed that SIN_1016759 was homologous gene of *PPO*, which encodes polyphenol oxidase and produce black pigments through the browning reaction in plant (Mayer, 2006). Therefore, we concluded that SIN_1016759 (*PPO*) might be the candidate gene that controlling seed coat color in sesame.

## Discussion

In this work, we obtained the draft genome of two representative landraces, “Baizhima” and “Mishuozhima,” based on next generation sequencing technologies and *de novo* assembly. Genome of the landraces offers an opportunity to comprehensively investigate the genome variations in the landraces of sesame at the nucleotide resolution. After a whole genome comparison of the landrace genomes and the reference genome, we succeeded to identify the first comprehensive catalog of SNPs and InDels among the samples, containing more than 1,800,000 variants. These genome variations are a source of genetic variability, generating new allelic variants during natural or artificial selection. Although the large-effect InDels in *PPO* genes have been validated, it should be noticed that the SNPs and InDels detected still needed further experimental validation.

This study confirms that a single genome of variety does not adequately represent the diversity contained within sesame. The availability of the “Baizhima” and “Mishuozhima” genome would help to identify variations in gene and nucleotide level. Because of the genetic bottleneck, genes might be lost during the domestication (Sang and Ge, 2013). Identification of new genes in multiple genomes is possible. New genes have been identified in different genome of rice and grape (Di Genova et al., 2014; Wang et al., 2015). Some lost genes might be identified in the sesame landrace genomes. And novel molecular markers could be designed for sesame genetic research and breeding. Rare SNPs and InDels are useful for DNA marker-assisted improvement and providing new information about how variation is distributed across the genomes (Ohmido et al., 2000). Benefiting from the high genome coverage sequencing, large sample of variation in low-copy have been identified.

The variable distribution of SNPs and InDels rates in sesame landraces genome has been reported in several other plant genomes (Lai et al., 2010; Arai-Kichise et al., 2011; Evans et al., 2013; Yadav et al., 2015). Some of the variations might come from the natural selection during domestication. It is thought that sesame were domesticated 5000 years ago, then moved along trade routes to quite different environment and underlined natural selection to adapted the conditions (Bedigian, 2003; Fuller, 2003). In addition, the regions of high SNPs and InDels density could correspond to regions containing genes under divergent selection, or recombination hot spots that result in elevated localized mutation rates (Lercher and Hurst, 2002). Genes for disease resistance or adaptation to the environment are often associated with recombination hot spots and highly various of the genes provide a selective advantage for crops (Lai et al., 2010; Evans et al., 2013). Alternatively, low density of SNPs and InDels in the linkage group regions indicates a large reduction of genetic variation and strong selective sweeps might occur in these regions. Selective sweep usually results from artificial selection of significant superior alleles for crops and rapid fixing of the superior alleles in modern varieties (Huang et al., 2012). For sesame breeders, knowledge of the nature of diversity in genomes, and the presence of low genetic diversity regions, could contribute to the design of breeding approaches that accelerate crop improvement.

To meet the quickly increasing labor cost of sesame cultivation and continuously increasing consumption of sesame, there is an enhancing effort to create mechanized cultivation varieties with super high yield. “Zhongzhi13” is the most widely grown varieties in Yangtze River Basin in the last 10 years (Wang et al., 2014b). But the high plant height and long mature period have prevented it to be a suitable variety for mechanized harvesting. Thus, plant height reducing, flowering early and yield increasing are important targets of the sesame genetic improvement. “Mishuozhima” is a typical semi-dwarf landrace with early flowering date. “Baizhima” is a much branched landrace with multi seeds in the capsules. Both of them could provide valuable alleles for genetic improvement of sesame varieties. Other superior traits that might confer advantages to “Zhongzhi13” of the two landraces include strong disease resistance, high sesamin content, and black coat of seed. Linkage mapping of several important traits have been carried out in the superior QTL seeking of sesame landraces (Zhang et al., 2013, 2014; Wu et al., 2014). However, plenty candidate genes were located in the QTL region and it is difficult to determine which one is the key gene. The identified SNPs and InDels in “Baizhima” and “Mishuozhima” genome could be used as molecular markers and help to improve the saturation of the genomic region where a QTL has been identified, which should reduce substantially the list of candidate genes in the QTLs. In addition, the availability of SNPs and InDels could provide valuable information to gene functional analysis and help in the identification of candidates genes related to traits of interest (Pan et al., 2008).

Seed coat color has been domesticated and selected significantly in sesame. Seed coat color of sesame in wild sesame is black and was domesticated to be light colors in landraces. Then in the modern varieties, more than 98% was white seed (Wei et al., 2015). It is reported that sesame seed coat color was controlled by several major QTLs (Zhang et al., 2013; Wang et al., 2016). In fact, besides the major QTL which *PPO* located, three other QTLs of sesame coat color were identified. Previous research indicated that seed coat color was affected by various pigments including flavonols, proanthocyanidin, and other phenolic relatives, like lignin and melanin (Yu, 2013). And the genes involved in flavonoid metabolism were cloned from *Arabidopsis* (Saito et al., 2013), rapeseed (Li X. et al., 2012), grape (Malacarne et al., 2016), and soybean (Yang et al., 2010). Thus, genes involved in the flavonoid synthesis might be the candidate genes of sesame coat color in the other identified QTLs. And the genome variations detected in these QTLs will provide valuable clues for future fine mapping and cloning of the genes of sesame seed coat color. Seed coat color significantly associated with oil content in sesame (Wei et al., 2015). The sesame seeds with light seed coat color tend to have higher oil content. Similar phenomenon is also found in the rapeseed seeds (Tang et al., 1997). *BrTT8* had been identified as the functional gene of yellow seed coat of rapeseed and the homolog gene of *BrTT8* in *Arabidopsis, TT8*, was reported to inhibit seed fatty acid accumulation by targeting several seed development regulators (Li X. et al., 2012; Chen et al., 2014). Therefore, we concluded that seed coat genes might affect the lipid synthesis of sesame and the cloning of seed coat gene might provide new strategy of oil content improvement for oilseed crops.

A large number of large-effect SNPs and InDels have been identified in the genes that related to plant development, disease resistant, flowering, and lipid metabolism. The identified large-effect genomic variations in sesame can be used in discovery and cloning of candidate genes related to the significant agronomic traits. And the variations in these genes could be easily converted into effective selection markers and used in the molecular marker assisted selection breeding of sesame.

## Conclusion

*De novo* genome sequencing of two representative sesame landraces, “Baizhima” and “Mishuozhima” had been completed and draft genome with contig N50 47 kb were obtained. Genome comparison of the landrace and modern variety genomes identified more than 1,800,000 SNPs and InDels, which were the source of agronomic traits variations in sesame. A large number of genomic variations related to important traits were also detected in the landrace genomes, which will provide valuable gene resources to sesame breeding. And the availability of the SNPs and InDels should accelerate the QTL mapping and gene cloning of sesame. Molecular markers developed from the SNPs and InDels could become a powerful tool in molecular assisted breeding. All of these will largely improve the efficiency of genetic improvement in sesame.

## Author contributions

XRZ and XW contributed to the design of this research. XW, XDZ, DL, RZ participated in the genome sequencing, QTL mapping, and qRT-PCR experiment. XW, JY, LW, YZ analyzed the data. XW wrote the manuscript.

## Funding

This work was funded by the National Natural Science Foundation of China (31401412), Agricultural Science and Technology Innovation Project of Chinese Academy of Agricultural Sciences (CAAS-ASTIP-2013-OCRI), and Core Research Budget of the Non-profit Governmental Research Institution (1610172014003).

### Conflict of interest statement

The authors declare that the research was conducted in the absence of any commercial or financial relationships that could be construed as a potential conflict of interest.

## Abbreviations

SNP(s): Single nucleotide polymorphism(s)
SSR marker: Simple sequence repeat marker
InDel(s): Insertion(s) and deletion(s)
SV(s): Structural variant(s)
CDS(s): Coding DNA sequence
qRT-PCR: Quantitative real time PCR
QTL: Quantitative trait loci
LGs: Linkage groups.

## References

[B1] Altschul S. F. Madden T. L. Schaffer A. A. Zhang J. Zhang Z. Miller W.. (1997). Gapped BLAST and PSI-BLAST: a new generation of protein database search programs. Nucleic Acids Res. 25, 3389–3402. 10.1093/nar/25.17.3389 9254694 PMC146917

[B2] Arai-Kichise Y. Shiwa Y. Nagasaki H. Ebana K. Yoshikawa H. Yano M.. (2011). Discovery of genome-wide DNA polymorphisms in a landrace cultivar of *Japonica* rice by whole-genome sequencing. Plant Cell Physiol. 52, 274–282. 10.1093/pcp/pcr003 21258067 PMC3037082

[B3] Baena-Gonzalez E. Rolland F. Thevelein J. M. Sheen J. (2007). A central integrator of transcription networks in plant stress and energy signalling. Nature 448, 938–942. 10.1038/nature06069 17671505

[B4] Barboza L. Effgen S. Alonso-Blanco C. Kooke R. Keurentjes J. J. B. Koornneef M.. (2013). *Arabidopsis* semidwarfs evolved from independent mutations in *GA20ox1*, ortholog to green revolution dwarf alleles in rice and barley. Proc. Natl. Acad. Sci. U.S.A. 110, 15818–15823. 10.1073/pnas.1314979110 24023067 PMC3785751

[B5] Bedigian D. (2003). Evolution of sesame revisited: domestication, diversity and prospects. Genet. Resour. Crop Evol. 50, 779–787. 10.1023/A:1025029903549

[B6] Bedigian D. (2010). Characterization of sesame (*Sesamum indicum* L.) germplasm: a critique. Genet. Resour. Crop Evol. 57, 641–647. 10.1007/s10722-010-9552-x

[B7] Cavanagh C. R. Chao S. Wang S. Huang B. E. Stephen S. Kiani S.. (2013). Genome-wide comparative diversity uncovers multiple targets of selection for improvement in hexaploid wheat landraces and cultivars. Proc. Natl. Acad. Sci. U.S.A. 110, 8057–8062. 10.1073/pnas.1217133110 23630259 PMC3657823

[B8] Chen M. Xuan L. Wang Z. Zhou L. Li Z. Du X.. (2014). *TRANSPARENT TESTA8* inhibits seed fatty acid accumulation by targeting several seed development regulators in *Arabidopsis*. Plant Physiol. 165, 905–916. 10.1104/pp.114.235507 24722549 PMC4044850

[B9] Chia J. M. Song C. Bradbury P. J. Costich D. de Leon N. Doebley J.. (2012). Maize HapMap2 identifies extant variation from a genome in flux. Nat. Genet. 44, 803–807. 10.1038/ng.2313 22660545

[B10] Di Genova A. Almeida A. M. Munoz-Espinoza C. Vizoso P. Travisany D. Moraga C.. (2014). Whole genome comparison between table and wine grapes reveals a comprehensive catalog of structural variants. BMC Plant Biol. 14:7. 10.1186/1471-2229-14-7 24397443 PMC3890619

[B11] Ding X. Wang L. Zhang Y. Li D. Gao Y. Wei W. (2013). Genetic variation and associated mapping for traits related to plant height constitutions in core collections of sesame (*Sesamum indicum* L.). Chin. J. Oil Crop Sci. 35, 262–270. 10.7505/j.issn.1007-9084.2013.03.006

[B12] Doebley J. F. Gaut B. S. Smith B. D. (2006). The molecular genetics of crop domestication. Cell 127, 1309–1321. 10.1016/j.cell.2006.12.006 17190597

[B13] El-Hamid Sayid El-Bramawy M. El-Hendawy S. Amin Shaban W. (2008). Assessing the suitability of morphological and phenological traits to screen sesame genotypes for *Fusarium wilt* and charcoal rot disease resistance. J. Plant Protect. Res. 48, 397–410. 10.2478/v10045-008-0049-y

[B14] Evans J. McCormick R. F. Morishige D. Olson S. N. Weers B. Hilley J.. (2013). Extensive variation in the density and distribution of DNA polymorphism in sorghum genomes. PLoS ONE 8:e79192. 10.1371/journal.pone.0079192 24265758 PMC3827139

[B15] Faostat (2015). Statistical Databases. Food and Agriculture Organization of the United Nations.

[B16] Felsenstein J. (1989). PHYLIP-phylogeny inference package (version 3.2). Cladistics 5, 163–166.

[B17] Fowler S. Lee K. Onouchi H. Samach A. Richardson K. Coupland G.. (1999). *GIGANTEA*: a circadian clock-controlled gene that regulates photoperiodic flowering in *Arabidopsis* and encodes a protein with several possible membrane-spanning domains. EMBO J. 18, 4679–4688. 10.1093/emboj/18.17.4679 10469647 PMC1171541

[B18] Fuller D. Q. (2003). Further evidence on the prehistory of sesame. Asian Agrihist. 7, 127–137.

[B19] Gutierrez E. Monteverde E. Quijada P. (1994). Inheritance of seed coat color and number of locules per capsule in three cultivars of sesame *Sesamun indicum* L. Agronomia Trop. 44, 513–527.

[B20] Hu J. Wang Y. Fang Y. Zeng L. Xu J. Yu H.. (2015). A rare allele of *GS2* enhances grain size and grain yield in rice. Mol. Plant 8, 1455–1465. 10.1016/j.molp.2015.07.002 26187814

[B21] Huang X. Feng Q. Qian Q. Zhao Q. Wang L. Wang A.. (2009). High-throughput genotyping by whole-genome resequencing. Genome Res. 19, 1068–1076. 10.1101/gr.089516.108 19420380 PMC2694477

[B22] Huang X. H. Kurata N. Wei X. H. Wang Z. X. Wang A. Zhao Q.. (2012). A map of rice genome variation reveals the origin of cultivated rice. Nature 490, 497–501. 10.1038/nature11532 23034647 PMC7518720

[B23] Ibarra-Laclette E. Lyons E. Hernandez-Guzman G. Perez-Torres C. A. Carretero-Paulet L. Chang T. H.. (2013). Architecture and evolution of a minute plant genome. Nature 498, 94–98. 10.1038/nature12132 23665961 PMC4972453

[B24] Jia Q. J. Zhang J. J. Westcott S. Zhang X. Q. Bellgard M. Lance R.. (2009). GA-20 oxidase as a candidate for the semidwarf gene *sdw1*/*denso* in barley. Funct. Integr. Genomic 9, 255–262. 10.1007/s10142-009-0120-4 19280236

[B25] Johnson L. A. Suleiman T. M. Lusas E. W. (1979). Sesame protein: a review and prospectus. J. Am. Oil Chem. Soc. 56, 463–468. 10.1007/BF02671542 395182

[B26] Kanu P. J. (2011). Biochemical analysis of black and white sesame seeds from China. Am. J. Biochem. Mol. Biol. 1, 145–157. 10.3923/ajbmb.2011.145.157

[B27] Krzywinski M. Schein J. Birol I. Connors J. Gascoyne R. Horsman D.. (2009). Circos: an information aesthetic for comparative genomics. Genome Res. 19, 1639–1645. 10.1101/gr.092759.109 19541911 PMC2752132

[B28] Kurtz S. Phillippy A. Delcher A. L. Smoot M. Shumway M. Antonescu C.. (2004). Versatile and open software for comparing large genomes. Genome Biol. 5:R12. 10.1186/gb-2004-5-2-r12 14759262 PMC395750

[B29] Lai J. S. Li R. Q. Xu X. Jin W. W. Xu M. L. Zhao H. N.. (2010). Genome-wide patterns of genetic variation among elite maize inbred lines. Nat. Genet. 42, 1027–1030. 10.1038/ng.684 20972441

[B30] Lercher M. J. Hurst L. D. (2002). Human SNP variability and mutation rate are higher in regions of high recombination. Trends Genet. 18, 337–340. 10.1016/S0168-9525(02)02669-0 12127766

[B31] Li C. Miao H. Wei L. Zhang T. Han X. Zhang H. (2014). Association mapping of seed oil and protein content in *Sesamum indicum* L. using SSR markers. PLoS ONE 9:e105757. 10.1371/journal.pone.0105757 25153139 PMC4143287

[B32] Li D. Liu W. Zhang Y. Wang L. Wei W. Gao Y. (2013). Identification method of drought tolerance and association mapping for sesame (*Sesamum indicum* L.). Acta Agron. Sin. 39, 1425–1433. 10.3724/SP.J.1006.2013.01425

[B33] Li S. C. Wang S. Q. Deng Q. M. Zheng A. P. Zhu J. Liu H. N.. (2012). Identification of genome-wide variations among three elite restorer lines for hybrid-rice. PLoS ONE 7:e30952. 10.1371/journal.pone.0030952 22383984 PMC3285608

[B34] Li X. Chen L. Hong M. Zhang Y. Zu F. Wen J.. (2012). A large insertion in bHLH transcription factor *BrTT8* resulting in yellow seed coat in *Brassica rapa*. PLoS ONE 7:e44145. 10.1371/journal.pone.0044145 22984469 PMC3439492

[B35] Lin T. Zhu G. T. Zhang J. H. Xu X. Y. Yu Q. H. Zheng Z.. (2014). Genomic analyses provide insights into the history of tomato breeding. Nat. Genet. 46, 1220–1226. 10.1038/ng.3117 25305757

[B36] Lisch D. (2013). How important are transposons for plant evolution? Nat. Rev. Genet. 14, 49–61. 10.1038/nrg3374 23247435

[B37] Luo R. Liu B. Xie Y. Li Z. Huang W. Yuan J.. (2012). SOAPdenovo2: an empirically improved memory-efficient short-read de novo assembler. Gigascience 1:18. 10.1186/2047-217X-1-18 23587118 PMC3626529

[B38] Malacarne G. Coller E. Czemmel S. Vrhovsek U. Engelen K. Goremykin V.. (2016). The grapevine *VvibZIPC22* transcription factor is involved in the regulation of flavonoid biosynthesis. J. Exp. Bot. 67, 3509–3522. 10.1093/jxb/erw181 27194742 PMC4892739

[B39] Mayer A. M. (2006). Polyphenol oxidases in plants and fungi: Going places? A review. Phytochemistry 67, 2318–2331. 10.1016/j.phytochem.2006.08.006 16973188

[B40] Ohmido N. Kijima K. Akiyama Y. de Jong J. H. Fukui K. (2000). Quantification of total genomic DNA and selected repetitive sequences reveals concurrent changes in different DNA families in indica and japonica rice. Mol. Gen. Genet. 263, 388–394. 10.1007/s004380051182 10821172

[B41] Pan C. Li A. Dai Z. Zhang H. Liu G. Wang Z. (2008). InDel and SNP markers and their applications in map-based cloning of rice genes. Rice Sci. 15, 251–258. 10.1016/S1672-6308(09)60001-9

[B42] Pathak N. Bhaduri A. Bhat K. V. Rai A. K. (2015). Tracking sesamin synthase gene expression through seed maturity in wild and cultivated sesame species–a domestication footprint. Plant Biol. (Stuttg). 17, 1039–1046. 10.1111/plb.12327 25754459

[B43] Pathirana R. (1994). Natural cross-pollination in sesame (*Sesamum indicum* L.). Plant Breed. 112, 167–170. 10.1111/j.1439-0523.1994.tb00665.x

[B44] Putterill J. Robson F. Lee K. Simon R. Coupland G. (1995). The *CONSTANS* gene of *Arabidopsis* promotes flowering and encodes a protein showing similarities to zinc-finger transcription factors. Cell 80, 847–857. 10.1016/0092-8674(95)90288-0 7697715

[B45] Rice P. Longden I. Bleasby A. (2000). EMBOSS: the European Molecular Biology Open Software Suite. Trends Genet. 16, 276–277. 10.1016/S0168-9525(00)02024-2 10827456

[B46] Saito K. Yonekura-Sakakibara K. Nakabayashi R. Higashi Y. Yamazaki M. Tohge T.. (2013). The flavonoid biosynthetic pathway in *Arabidopsis*: structural and genetic diversity. Plant Physiol. Biochem. 72, 21–34. 10.1016/j.plaphy.2013.02.001 23473981

[B47] Salamov A. A. Solovyev V. V. (2000). Ab initio gene finding in *Drosophila* genomic DNA. Genome Res. 10, 516–522. 10.1101/gr.10.4.516 10779491 PMC310882

[B48] Sang T. Ge S. (2013). Understanding rice domestication and implications for cultivar improvement. Curr. Opin. Plant Biol. 16, 139–146. 10.1016/j.pbi.2013.03.003 23545218

[B49] Shahidi F. Liyana-Pathirana C. M. Wall D. S. (2006). Antioxidant activity of white and black sesame seeds and their hull fractions. Food Chem. 99, 478–483. 10.1016/j.foodchem.2005.08.009

[B50] Somers D. E. Schultz T. F. Milnamow M. Kay S. A. (2000). *ZEITLUPE* encodes a novel clock-associated PAS protein from *Arabidopsis*. Cell 101, 319–329. 10.1016/S0092-8674(00)80841-7 10847686

[B51] Song H. R. Song J. D. Cho J. N. Amasino R. M. Noh B. Noh Y. S. (2009). The RNA binding protein *ELF9* directly reduces *SUPPRESSOR OF OVEREXPRESSION OF CO1* transcript levels in arabidopsis, possibly via nonsense-mediated mRNA decay. Plant Cell 21, 1195–1211. 10.1105/tpc.108.064774 19376936 PMC2685614

[B52] Spielmeyer W. Ellis M. H. Chandler P. M. (2002). Semidwarf (sd-1), “green revolution” rice, contains a defective gibberellin 20-oxidase gene. Proc. Natl. Acad. Sci. U.S.A. 99, 9043–9048. 10.1073/pnas.132266399 12077303 PMC124420

[B53] Springer N. M. Ying K. Fu Y. Ji T. M. Yeh C. T. Jia Y.. (2009). Maize inbreds exhibit high levels of copy number variation (CNV) and presence/absence variation (PAV) in genome content. PLoS Genet. 5:e1000734. 10.1371/journal.pgen.1000734 19956538 PMC2780416

[B54] Sun J. Yan T. Gao D. Yang G. Xu G. Liu W. (2015). Breeding characteristics of *Sesamum indicum* L. I: estimations of spontaneous outcrossing rates. Chin. J. Oil Crop Sci. 37, 462–466. 10.7505/j.issn.1007-9084.2015.04.005

[B55] Tang Z. L. Li J. N. Zhang X. K. Chen L. Wang R. (1997). Genetic variation of yellow-seeded rapeseed lines (*Brassica napus* L.) from different genetic sources. Plant Breed. 116, 471–474. 10.1111/j.1439-0523.1997.tb01033.x

[B56] Wang H. Deng X. W. (2004). Phytochrome signaling mechanism. Arabidopsis Book 3, e0074.e0071. 10.1199/tab.0074.1 22303226 PMC3243403

[B57] Wang L. Han X. Zhang Y. Li D. Wei X. Ding X.. (2014a). Deep resequencing reveals allelic variation in *Sesamum indicum*. BMC Plant Biol. 14:225. 10.1186/s12870-014-0225-3 25138716 PMC4148021

[B58] Wang L. Xia Q. Zhang Y. Zhu X. Zhu X. Li D.. (2016). Updated sesame genome assembly and fine mapping of plant height and seed coat color QTLs using a new high-density genetic map. BMC Genomics 17:31. 10.1186/s12864-015-2316-4 26732604 PMC4702397

[B59] Wang L. Yu S. Tong C. Zhao Y. Liu Y. Song C.. (2014b). Genome sequencing of the high oil crop sesame provides insight into oil biosynthesis. Genome Biol. 15:R39. 10.1186/gb-2014-15-2-r39 24576357 PMC4053841

[B60] Wang Y. X. Xiong G. S. Hu J. Jiang L. Yu H. Xu J.. (2015). Copy number variation at the *GL7* locus contributes to grain size diversity in rice. Nat. Genet. 47, 944–948. 10.1038/ng.3346 26147619

[B61] Wei W. Zhang Y. Lu H. Li D. Wang L. Zhang X. (2013). Association analysis for quality traits in a diverse panel of Chinese sesame (*Sesamum indicum* L.) germplasm. J. Integr. Plant Biol. 55, 745–758. 10.1111/jipb.12049 23570323

[B62] Wei X. Liu K. Zhang Y. Feng Q. Wang L. Zhao Y.. (2015). Genetic discovery for oil production and quality in sesame. Nat. Commun. 6, 8609. 10.1038/ncomms9609 26477832 PMC4634326

[B63] Wei X. Wang L. Zhang Y. Qi X. Wang X. Ding X.. (2014). Development of simple sequence repeat (SSR) markers of sesame (*Sesamum indicum*) from a genome survey. Molecules 19, 5150–5162. 10.3390/molecules19045150 24759074 PMC6270694

[B64] Weiss E. A. (2000). Oilseed Crops. Oxford: Blackwell Science.

[B65] Wu K. Liu H. Y. Yang M. M. Tao Y. Ma H. H. Wu W. X.. (2014). High-density genetic map construction and QTLs analysis of grain yield-related traits in sesame (*Sesamum indicum* L.) based on RAD-Seq techonology. BMC Plant Biol. 14:274. 10.1186/s12870-014-0274-7 25300176 PMC4200128

[B66] Yadav C. B. Bhareti P. Muthamilarasan M. Mukherjee M. Khan Y. Rathi P.. (2015). Genome-wide SNP identification and characterization in two soybean cultivars with contrasting *mungbean yellow mosaic india virus* disease resistance traits. PLoS ONE 10:e0123897. 10.1371/journal.pone.0123897 25875830 PMC4395324

[B67] Yang K. Jeong N. Moon J. K. Lee Y. H. Lee S. H. Kim H. M.. (2010). Genetic analysis of genes controlling natural variation of seed coat and flower colors in soybean. J. Hered. 101, 757–768. 10.1093/jhered/esq078 20584753

[B68] Yu C. Y. (2013). Molecular mechanism of manipulating seed coat coloration in oilseed *Brassica* species. J. Appl. Genet. 54, 135–145. 10.1007/s13353-012-0132-y 23329015

[B69] Yue W. Wei L. Zhang T. Li C. Miao H. Zhang H. (2013). Genetic diversity and population structure of germplasm resources in sesame (*Sesamum indicum* L.) by SSR markers. Acta Agron. Sin. 38, 2286–2296. 10.3724/SP.J.1006.2012.2286

[B70] Zamir D. (2001). Improving plant breeding with exotic genetic libraries. Nat. Rev. Genet. 2, 983–989. 10.1038/35103590 11733751

[B71] Zeven A. C. (1998). Landraces: a review of definitions and classifications. Euphytica 104, 127–139. 10.1023/a:1018683119237

[B72] Zhang H. Miao H. Wei L. Li C. Zhao R. Wang C. (2013). Genetic analysis and QTL mapping of seed coat color in sesame (*Sesamum indicum* L.). PLoS ONE 8:e63898. 10.1371/journal.pone.0063898 23704951 PMC3660586

[B73] Zhang Y. Wang L. Li D. Gao Y. Lu H. Zhang X. (2014). Mapping of sesame waterlogging tolerance QTL and identification of excellent waterlogging tolerant germplasm. China Agric. Sci. 45, 2580–2591. 10.3864/j.issn.0578-1752.2014.03.002

[B74] Zhang Y. Wang L. Li D. Wei W. Gao Y. Zhang X. (2012). Association mapping of sesame (*Sesamum indicum* L.) resistance to macrophomina phaseolina and identification of resistant accessions. Sci. Agric. Sin. 45, 2580–2591. 10.3864/j.issn.0578-1752.2012.13.003

[B75] Zhou Z. Jiang Y. Wang Z. Gou Z. Lyu J. Li W.. (2015). Resequencing 302 wild and cultivated accessions identifies genes related to domestication and improvement in soybean. Nat. Biotechnol. 33, 408–414. 10.1038/nbt.3096 25643055

[B76] Zhu J. K. (2002). Salt and drought stress signal transduction in plants. Annu. Rev. Plant Biol. 53, 247–273. 10.1146/annurev.arplant.53.091401.143329 12221975 PMC3128348

[B77] Zuo W. Chao Q. Zhang N. Ye J. Tan G. Li B.. (2015). A maize wall-associated kinase confers quantitative resistance to head smut. Nat. Genet. 47, 151–157. 10.1038/ng.3170 25531751

