# Supplementary figures and images for: Identification of Sesame Genomic Variations from Genome Comparison of Landrace and Variety
Source: Front Plant Sci. 2016 Aug 3;7:1169. doi: 10.3389/fpls.2016.01169 (PMC4971434; doi:10.3389/fpls.2016.01169)

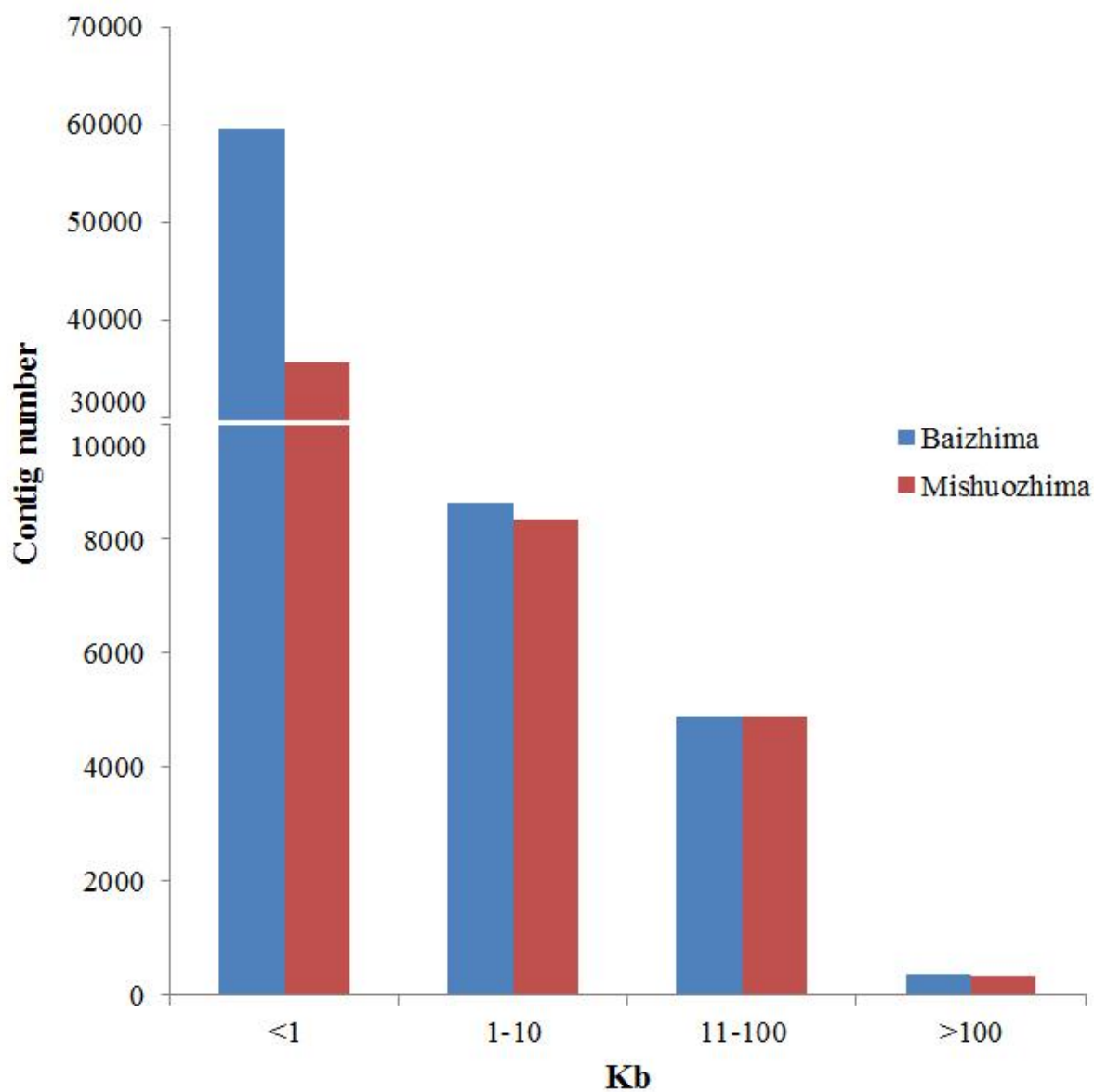

**Supplementary Figure S1 Length of contigs in assembled genomes of ‘Baizhima’ and ‘Mishuozhima’.**

Supplement: Supplementary file 13 [file Image1.PDF]
