# Supplementary material for: Identification of Sesame Genomic Variations from Genome Comparison of Landrace and Variety
Source: Front Plant Sci. 2016 Aug 3;7:1169. doi: 10.3389/fpls.2016.01169 (PMC4971434; doi:10.3389/fpls.2016.01169)
Supplement: Supplementary file 14 [file Image2.PDF]

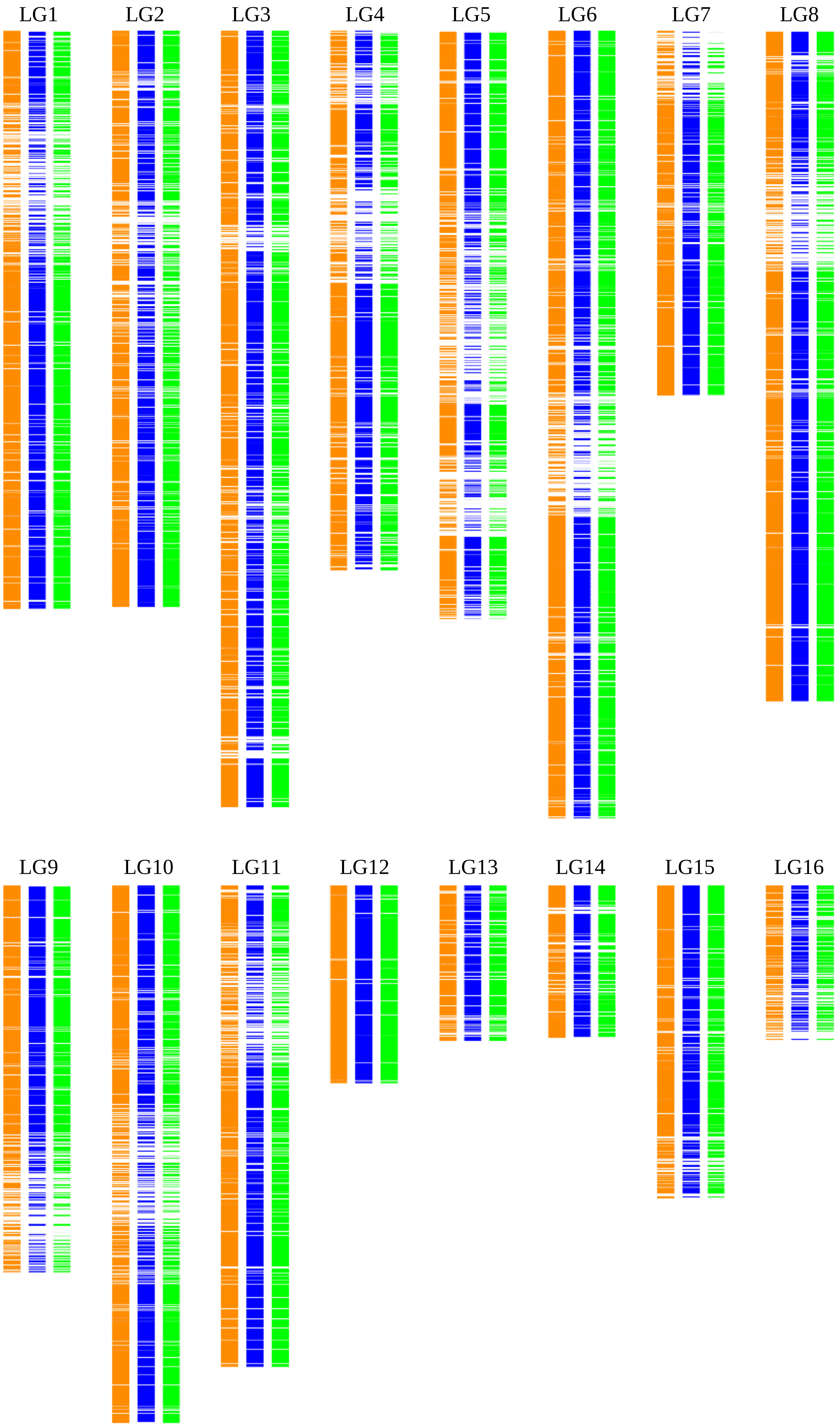

Supplementary Figure 2 Gene alignment of the three genomes. Yellow, blue and green indicate ‘Zhongzhi13’, ‘Baizhima’ and ‘Mishuozhima’, respectively.
