# Supplementary material for: Identification of Sesame Genomic Variations from Genome Comparison of Landrace and Variety
Source: Front Plant Sci. 2016 Aug 3;7:1169. doi: 10.3389/fpls.2016.01169 (PMC4971434; doi:10.3389/fpls.2016.01169)
Supplement: Supplementary file 15 [file Image3.PDF]

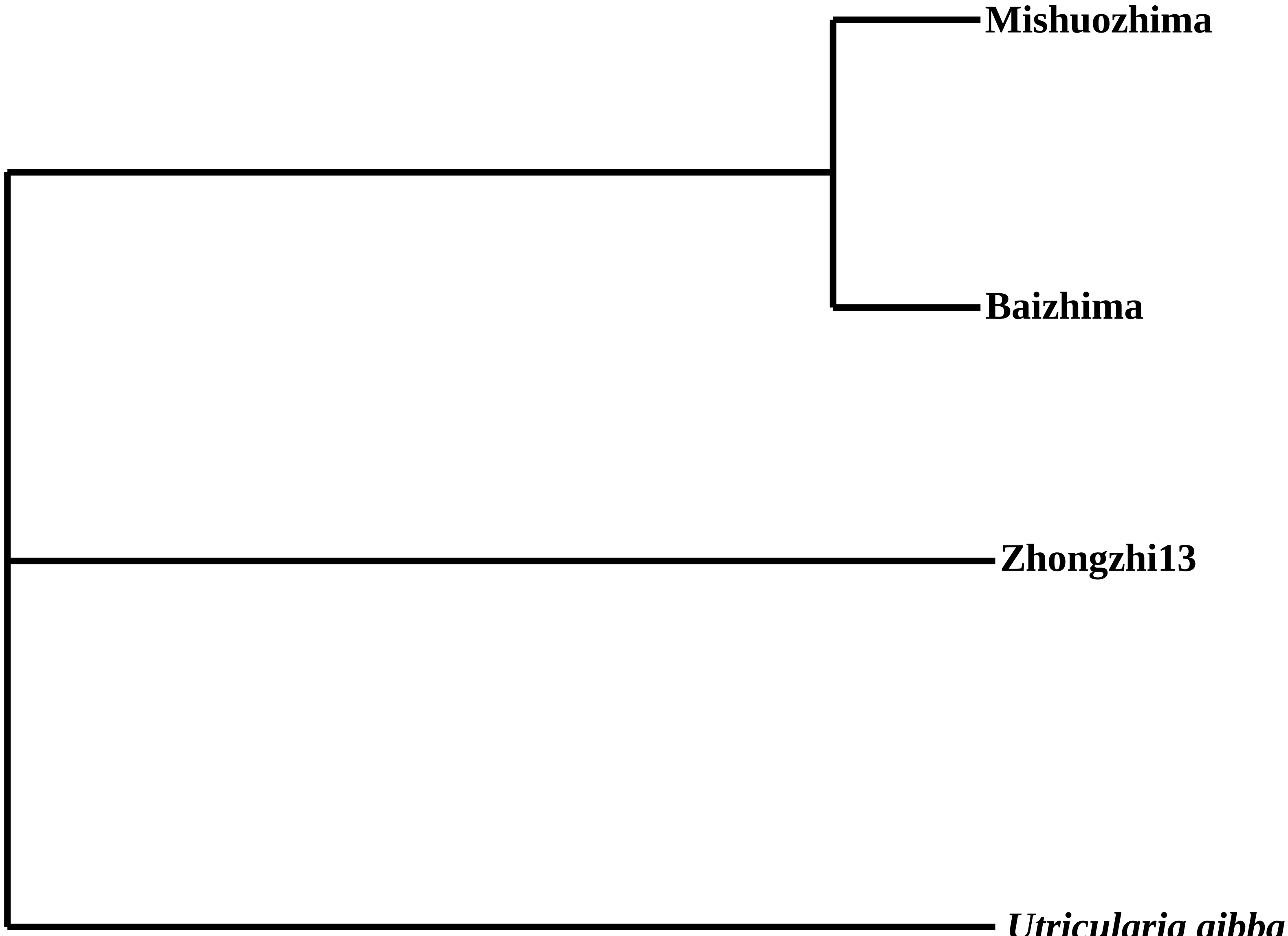

Supplementary Figure 3 Phylogenetic tree of the accessions. *Utricularia gibba* was used as out-group.
