# Supplementary material for: Identification of Sesame Genomic Variations from Genome Comparison of Landrace and Variety
Source: Front Plant Sci. 2016 Aug 3;7:1169. doi: 10.3389/fpls.2016.01169 (PMC4971434; doi:10.3389/fpls.2016.01169)
Supplement: Supplementary file 16 [file Image4.PDF]

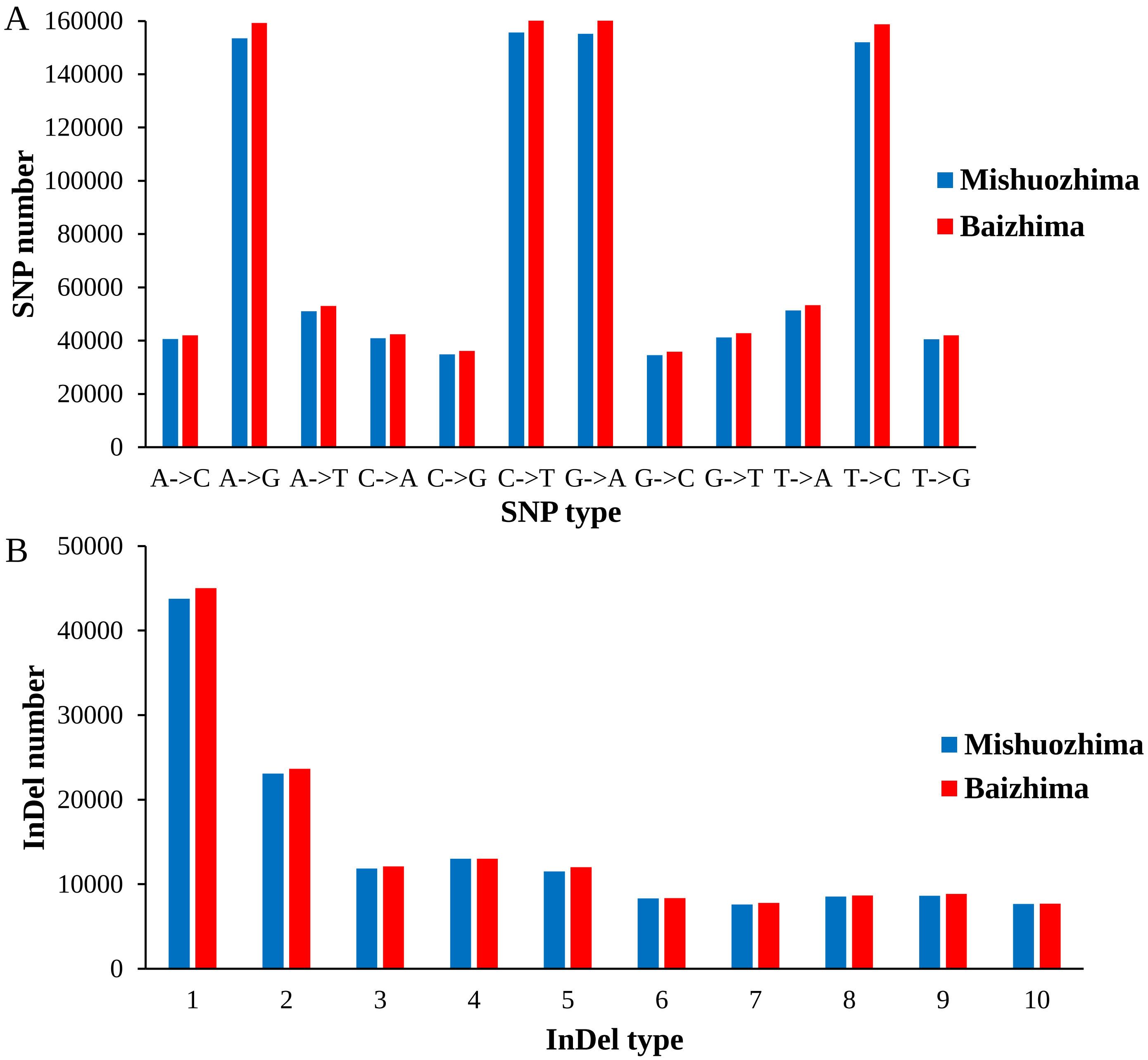

Supplementaty Figure 4 SNP and InDel types in in 'Baizhima' and 'Mishuozhima' genomes. (A) SNPs; (B) InDels, 1-10 bp.
