# Supplementary material for: Identification of Sesame Genomic Variations from Genome Comparison of Landrace and Variety
Source: Front Plant Sci. 2016 Aug 3;7:1169. doi: 10.3389/fpls.2016.01169 (PMC4971434; doi:10.3389/fpls.2016.01169)
Supplement: Supplementary file 17 [file Image5.PDF]

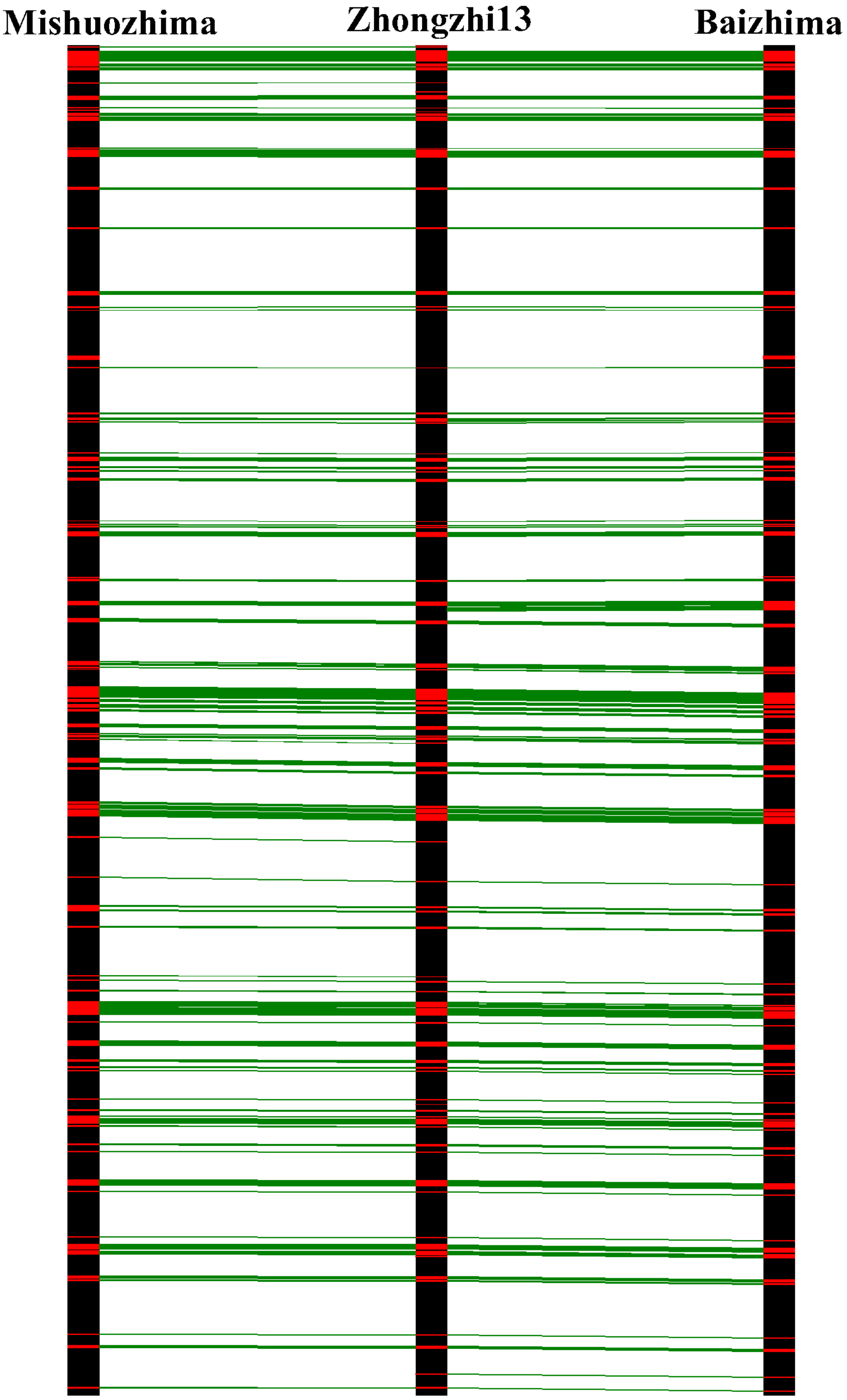

Supplementary Figure 5 Transposons alignment of the genomes of ‘Baizhima’, ‘Mishuozhima’ and ‘Zhongzhi13’. Red indicate transposons in the genomes. These regions are listed in Table S6.
