# Supplementary material for: Identification of Sesame Genomic Variations from Genome Comparison of Landrace and Variety
Source: Front Plant Sci. 2016 Aug 3;7:1169. doi: 10.3389/fpls.2016.01169 (PMC4971434; doi:10.3389/fpls.2016.01169)
Supplement: Supplementary file 19 [file Image7.PDF]

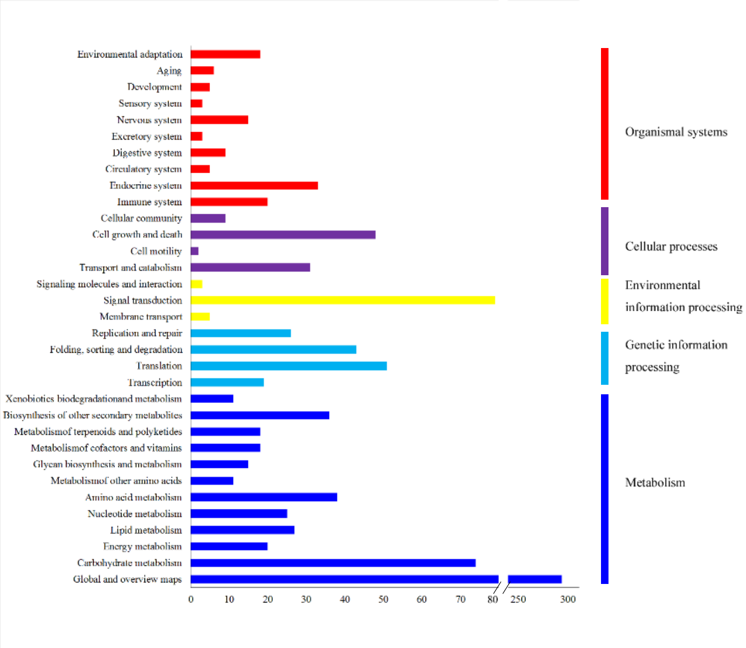

Supplementary Figure S7 KEGG pathway enrichment of the dN/dS > 1 genes in ‘Baizhima’ and ‘Mishuozhima’ genomes.
