# Supplementary material for: Identification of Sesame Genomic Variations from Genome Comparison of Landrace and Variety
Source: Front Plant Sci. 2016 Aug 3;7:1169. doi: 10.3389/fpls.2016.01169 (PMC4971434; doi:10.3389/fpls.2016.01169)
Supplement: Supplementary file 20 [file Image8.PDF]

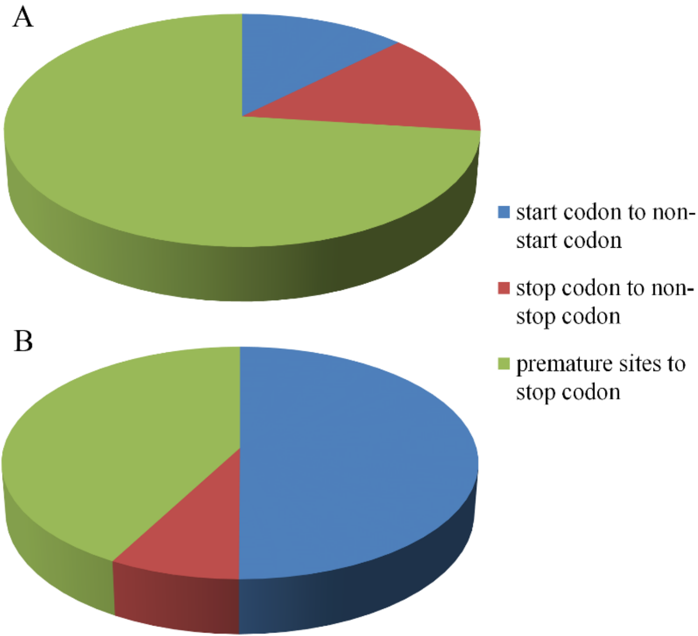

Supplementary Figure S8 Proportions of genes containing large-effect SNPs. (A) Baizhima; (B) Mishuozhima.
